# Diabetic gastroparesis: pathophysiology and impact on insulin timing choices

**DOI:** 10.1007/s12020-026-04693-6

**Published:** 2026-06-23

**Authors:** Francesco Galeano, Andrea Tumminia, Vittorio Oteri, Rosario Le Moli, Tommaso Piticchio, Sabrina Scilletta, Maurizio Di Marco, Antonino Di Pino, Francesco Frasca

**Affiliations:** 1 Department of Clinical and Experimental Medicine, Endocrinology Section, Garibaldi-Nesima Hospital, University of Catania, Catania, 95122 CT Italy; 2 Endocrine Unit, Garibaldi-Nesima Hospital, Catania, 95122 CT Italy; 3 Department of Medicine and Surgery, University Kore of Enna, Enna, 94100 EN Italy; 4 Unit of Diabetology, Metabolic and Endocrine Diseases, “Cannizzaro” Emergency Hospital, Catania, Italy; 5 Department of Clinical and Experimental Medicine, Internal Medicine, Garibaldi- Nesima Hospital, University of Catania, Catania, 95122 CT Italy

**Keywords:** Diabetic gastroparesis, Diabetic autonomic neuropathy, Insulin therapy, Carbohydrate absorption, Glycemic control

## Abstract

Diabetic gastroparesis (DGP) is a chronic and frequently underrecognized complication of diabetes mellitus, characterized by delayed gastric emptying in the absence of mechanical obstruction. It represents a clinically significant manifestation of diabetic autonomic neuropathy and arises from a complex interplay of sustained hyperglycemia, oxidative and nitrosative stress, advanced glycation end-product accumulation, impaired insulin/IGF-1 signaling, lipid dysmetabolism, and inflammatory activation. These mechanisms converge to damage autonomic and enteric neurons, reduce interstitial cells of Cajal density, and disrupt gastric smooth muscle function, ultimately leading to impaired and unpredictable gastric motility. Beyond gastrointestinal symptoms, DGP profoundly affects metabolic control. The altered timing and variability of gastric emptying disturb the physiological coordination between nutrient absorption and insulin pharmacokinetics, generating a temporal mismatch that predisposes patients to early postprandial hypoglycemia followed by late hyperglycemia. This pattern increases glycemic variability, complicates insulin dose adjustment, and contributes to oxidative stress and vascular risk. In this narrative review, we examine the current understanding of DGP pathophysiology and its clinical implications for insulin therapy. We discuss diagnostic strategies, nutritional interventions, and pharmacological treatments, with particular attention to insulin timing modulation. We also analyze the emerging role of diabetes technologies, including continuous glucose monitoring and automated insulin delivery systems, as well as future perspectives such as glucose-responsive insulins and regenerative approaches targeting gastric neuromuscular dysfunction. A mechanism-based, personalized therapeutic strategy may improve metabolic stability and patient outcomes.

**Supplementary Information:**

The online version contains supplementary material available at 10.1007/s12020-026-04693-6.

## Introduction

Diabetic autonomic neuropathy (DAN) is a chronic and multifactorial complication of diabetes, characterized by damage to autonomic nerve fibers, ultimately leading to multisystem dysfunction involving cardiovascular, gastrointestinal, genitourinary and sudomotor systems, as well as certain special sensory functions. DAN contributes to increased morbidity healthcare utilization, and reduced quality of life [1, 2].

Among DAN manifestations, gastrointestinal involvement holds particular relevance because of its impact on metabolic control, including the timing of postprandial glucose peaks. Diabetic gastroparesis (DGP), defined as “a motility disorder characterized by symptoms and objective documentation of delayed gastric emptying (GE) of solid food without mechanical obstruction” [3], typically confirmed by gastric emptying scintigraphy [4], presents with symptoms such as early satiety, postprandial fullness, bloating, nausea, vomiting, and abdominal discomfort. These symptoms can significantly impair dietary intake, nutritional status, and daily functioning, besides increasing hospitalization, and healthcare costs [4–6]. Delayed and erratic gastric emptying disrupts the synchronization between carbohydrate absorption and insulin action, creating a major challenge for glycemic management. DGP is estimated to affect 5% − 12% of individuals with long-term diabetes, in cohorts with systematic gastric emptying evaluation (symptom-based criteria plus objective testing) [7, 8], although epidemiological figures vary widely due to different diagnostic criteria and assessment methods. In contrast, large administrative databases report much lower prevalence, capturing only clinically coded cases without objective testing. For example, a large study conducted in 2020 on more than 43 million medical records in the United States reported that only 0.16% of patients had a documented diagnosis of gastroparesis [9, 10]. When analyzing the diabetic population specifically, the prevalence was 4.6% in patients with type 1 diabetes (T1D) and 1.3% in patients with type 2 diabetes (T2D) in a large US cohort relying on clinical diagnosis recorded in electronic medical records, without systematic requirement for gastric emptying scintigraphy [8]. In contrast, studies applying stricter criteria for “definite gastroparesis”, show that diabetic etiology represents the leading cause of gastroparesis among identified etiologies, accounting for approximately 57.4% of all reported cases in large national claims databases [10]. Risk correlates include long disease duration [8, 11, 12], poor glycemic control [3, 13], presence of other diabetic complications (especially peripheral and autonomic neuropathy) [4, 13], prior bariatric surgery [14, 15], and female sex [16, 17], while psychological factors (anxiety, depression, and somatization) may modulate symptom severity despite similar objective findings [4, 18–20]. Despite these insights, the natural history of DGP remains incompletely characterized: some patients show progressive worsening of symptoms and GE delay, others exhibit periods of apparent regression or fluctuation, and the overall temporal behaviour of symptom burden over years is poorly understood. Moreover, changes in symptom intensity do not always parallel objective GE measurements, highlighting the complex interplay between pathophysiological derangements and central processing of visceral signals [14, 21–23]. Despite its clinical relevance, DGP remains underrecognized because of nonspecific symptomatology overlapping with functional dyspepsia or gastroesophageal reflux disease (GERD) [24].

Although the clinical presentation of DGP is well characterized, its pathogenesis is multifactorial. involving metabolic, neuronal, vascular, and inflammatory mechanism, induced by chronic diabetes [25], that converge to alter gastric motor function and nutrient absorption.

The following section outlines the current understanding of the pathophysiological processes driving this disorder, forming the basis on which new therapeutic strategies, including technology-driven approaches to insulin delivery, are being developed.

## Methods

This narrative review searched the MEDLINE database via PubMed up to the 14 of December 2025, combining terms referring to diabetic gastroparesis, diabetes autonomic and enteric neuropathy, gastric motor dysfunction, and disorders of gastric emptying. The full search strategy is available: in Appendix S1.

The reference lists of the included articles and previous literature reviews on the topic were reviewed for further identification of potentially relevant studies.

**Fig. 1:**
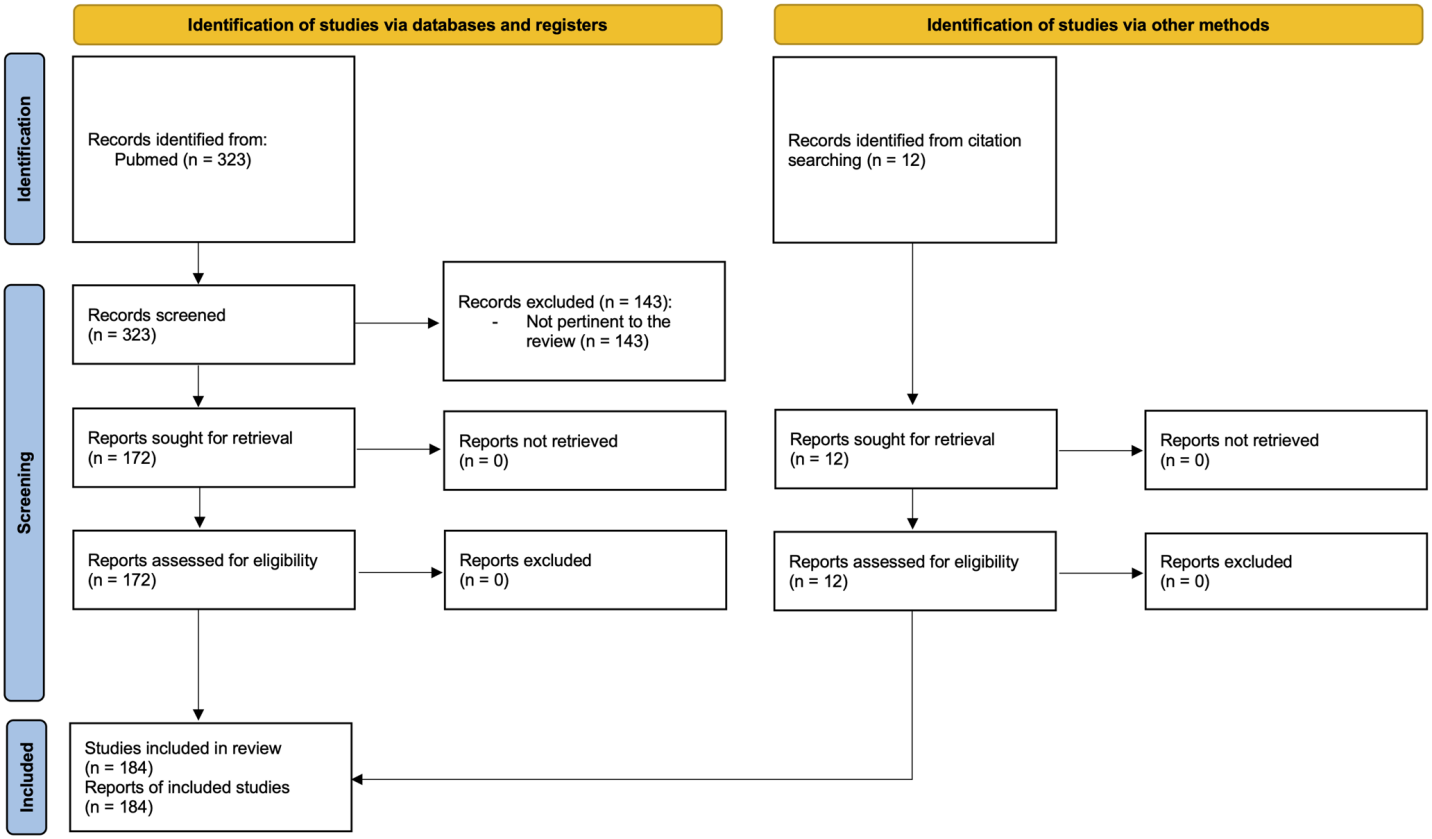
PRISMA flow diagram of study selection

Eligible studies included those investigating the relationships between the diabetic gastroparesis, autonomic neuropathy, gastric dysmotility, and their clinical and metabolic consequences in patients with diabetes, selected according to relevance and study quality rather than through formal meta-analysis or systematic review methodology. Given the narrative nature of this review, no formal risk-of-bias assessment was performed. We also excluded studies in which data were not accessible, missing, without an available full text, or not well reported. All data were extracted from articles’ text, tables, and figures using the Population, Intervention, Comparison, and Outcome (PICO) framework [26] and included at least the following: title, authors, year of publication, study design, study population, outcomes, and main results. The PRISMA flow diagram (Fig. 1) is provided for transparency of study identification but does not imply a systematic review methodology.

## Pathophysiology

The pathogenesis of DGP involves interconnected nervous, cellular, muscular, microvascular, and metabolic–inflammatory mechanisms rather than a single causal pathway. Sustained hyperglycemia represents the principal driver; however, disease duration, chronic glycemic variability, and acute hyperglycemia episodes also contribute independently to neural and motor disfunction, resulting in impaired motility and delayed gastric emptying [27–31].

**Fig. 2:**
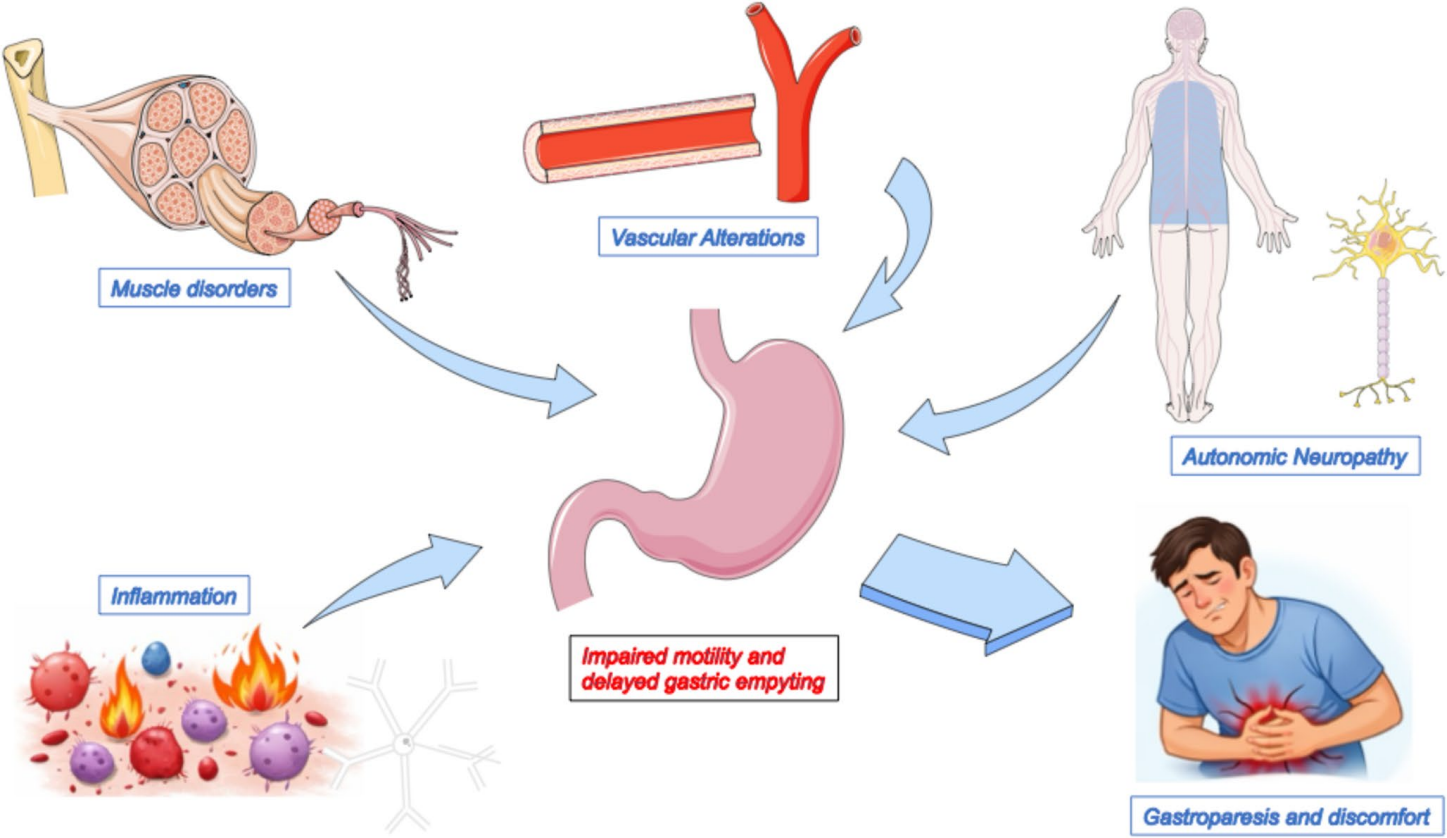
Pathogenesis of diabetic gastroparesis

Figure 2 provides a schematic integrative overview of these converging mechanisms and their interactions.

### Oxidative and nitrosative stress

Excess intracellular glucose promotes mitochondrial overactivity, leading to an increased formation of reactive oxygen and nitrogen species (ROS, RNS) [32]. These contribute to inflammatory microangiopathy and local inflammatory signaling promoting further damage to nerves, Cajal Cells (ICCs), and smooth muscle, impairing inhibitory neurotransmission and peristaltic rhythm generation [25]. It is therefore not surprising that inflammatory infiltrates have been documented in diabetic patients in the esophagus, supporting the concept of diffuse gastrointestinal involvement within diabetic autonomic neuropathy rather than isolated gastric disease.

### Advanced glycation and impaired antioxidant defenses

Chronic hyperglycemia enhances formation of advanced glycation end-products (AGEs), which bind to specific receptors (RAGE) and activate inflammatory pathways. Activation of the polyol pathway further increases oxidative stress, damaging neurons and endothelial cells and reducing blood supply to neural tissues [33–37].

### Lipid dysmetabolism and insulin resistance

In addition to hyperglycemia, alterations in lipid metabolism also play a central role in the pathogenesis of DAN. Low-density lipoproteins (LDL) can undergo oxidation and glycation processes, amplifying the oxidative stress already triggered by excess glucose [38].

Insulin resistance has direct effects on gastrointestinal function [39] : insulin acts as a trophic factor for neurons, promoting their survival and supporting nerve transmission. Its deficiency or reduced biological efficacy, contributes to neuronal damage and impaired gastric motility [40, 41].

### Autonomic and enteric neuropathy

Extrinsic vagal efferent pathways and intrinsic enteric nerves coordinate gastric accommodation, antral contraction and pyloric relaxation [25]. Damage to these pathways results in alteration of impulse tramission [28] with loss of gastric tone, reduced contractility and impaired coordination of peristaltic waves, slowing gastric emptying [25, 29, 30].

In addition, structural and functional alterations in gastric smooth muscle may contribute: in the gastric fundus, impaired inhibitory neurotransmission is accompanied by reduced tonic contraction because of defective muscle contractility, whereas in the antrum both cholinergic neuromuscular transmission and smooth muscle strength are altered [19, 23, 42, 43]. Moreover, advanced diabetic gastroparesis can be associated with smooth-muscle degeneration, fibrosis, collagen deposition, and ultrastructural abnormalities such as thickened basal lamina, changes that likely contribute to a hypocontractile phenotype and delayed gastric emptying [23, 42–45]. Importantly, these myogenic changes may occur in parallel with neuronal injury, supporting the concept of a partly independent muscular pathway in the disease process [42].

### Gut microbiota and DGP

Alterations in gut microbial composition may contribute to low-grade inflammation, barrier disfunction, and altered gut-brain signaling, influencing both gastric motility and symptom perception [46–48]. These microbiota-related changes may also help explain the frequent overlap between upper and lower gastrointestinal symptoms, including the coexistence of constipation and gastroparetic complaints [43, 48]. Notably, constipation is present in approximately one‑third of patients with gastroparesis and can exacerbate gastric symptoms by altering intestinal transit and generating retrograde feedback signals. This integrated, pan‑enteric perspective underlines the importance of evaluating and managing gastrointestinal motility as a whole, and may lead to more effective interventions for both gastric and colonic symptoms [49].

### “Cajalopathy”: dysfunction of interstitial cells of cajal (ICCs)

Another key player in the pathophysiology of gastroparesis is the ICCs. These cells act as gastrointestinal pacemakers, coordinating the rhythmic contractions necessary for the transit of gastric contents [50, 51].

In diabetes, chronic hyperglycemia, and reduced trophic factors, in particular the reduction of KIT/stem cell factor (SCF), cause the loss or dysfunction of ICCs [52], leading to dysregulated gastric emptying [4, 19, 53]. This condition, often referred to as “cajalopathy”, is considered the most common alteration found in both diabetic and idiopathic gastroparesis [54]. Oxidative stress, resulting from increased ROS production under chronic hyperglycemia, leads to direct damage to ICCs and neuronal terminals [23, 55, 56], also the enzyme HO-1 reduced expression promotes ICC loss and the development of gastroparesis [57]. In diabetes, insulin and IGF-1 deficiency causes muscle atrophy and reduced SCF production, leading to ICC depletion and impaired neuromuscular transmission [52–60].

The reduction in ICCs interrupts functional communication between enteric neurons and smooth muscle, generating irregular slow waves and variable responses to interventions such as gastric electrical stimulation [54]. This circular interaction, in which nerves and pacemaker cells negatively influence each other, is one of the main mechanisms underlying the marked delay in gastric emptying typical of diabetic gastroparesis.

## Diagnosis

DPG cannot be diagnosed on the basis of symptoms alone, as its clinical presentation soverlaps with functional dyspepsia or GERD [61]. For this reason, the diagnostic pathway requires instrumental documentation of delayed gastric emptying in the absence of mechanical obstruction, following an appropriate evaluation to rule out structural causes through endoscopy or cross-sectional imaging [3, 4, 14, 61].

Accurate diagnosis requires a structured assessment of symptom severity and response to therapy. The Gastroparesis Cardinal Symptom Index (GCSI) and the Patient Assessment of Gastrointestinal Disorders–Symptom Severity Index (PAGI‑SYM) provide validated, quantitative measures of gastroparetic symptoms and are useful for screening and monitoring treatment response in clinical practice and trials [62, 63].

Gastric emptying scintigraphy (GES) is recognized as the gold standard for the diagnosis of gastroparesis. The recommended protocol involves the ingestion of a standardized radiolabeled solid meal with imaging at 0, 1, 2, and 4 h [14, 61]. Quantitative interpretation is based on established retention thresholds at each time point: >90% retention at 1 h, > 60% at 2 h, > 10% at 4 h are diagnostic for delayed gastric emptying. The 4-hour acquisition is considered the most sensitive and specific parameter and is used to stratify severity [14]. Despite its strengths, GES presents limitations: protocols variability, limited availability, and radiation exposure [14, 61, 64].

Non-invasive alternatives include the 13 C breath test whicj measures exhaled ¹³CO₂ following ingestion of a meal containing a stable isotope–labeled substrate [14, 61, 65, 66]. It indirectly reflects solid meal gastric emptying, providing a radiation-free option for repeated measurements and for use in children, pregnant women, and breastfeeding women [67, 68]. Concordance with scintigraphy is generally good, particularly for identifying significant delays in motility [3, 14, 66]. However, results can be influenced by extra gastric factors such as liver function, pulmonary disease, and small-intestinal absorption kinetics [3, 14, 66, 69, 70].

Advanced techniques, including antroduodenal‑jejunal manometry [71–75], wireless motility capsule [76–79], gastric ultrasound [80–82], and high‑resolution electrogastrography (EGG) [83–87] may provide additional pathophysiological insight, particularly in complex or mixed‑pattern cases. However, their use remains largely confined to specialized centers or research settings.

Given that gastric emptying is a highly dynamic influenced by hyperglycemia, pharmacological agents, recent dietary intake, and neurohormonal fluctuations, strict pre-test standardization is essential regardless of the diagnostic modality employed. Failure to control these variables may lead to substantial intra-individual variability and misclassification of gastric emptying status, particularly in patients with diabetes, in whom gastric motor function may fluctuate over time [3, 14]. Moreover, no single diagnostic technique fully captures the heterogeneous and multifactorial pathophysiology of diabetic gastroparesis.

By delineating not only the presence but also the pattern and variability of gastric dysmotility, advanced diagnostic approaches may help identify patients at greatest risk for mismatches between nutrient absorption and insulin action, thereby setting the stage for a more individualized and physiologically informed approach to insulin therapy.

Table 1 summarizes the step‑by‑step diagnostic approach to diabetic gastroparesis, with particular reference to first‑line clinical and non‑invasive assessments, second‑line objective tests of gastric emptying, and selected advanced motility studies available in specialized centers.

**Table 1:** Step‑by‑step diagnostic approach to diabetic gastroparesis

Step / Modality	Role in the diagnostic pathway	Main advantages	Main limitations
First-level diagnostic assessments			
Clinical history and symptom assessment (including GCSI/PAGY-SYM)	Initial evaluation ad screening; stratification of symptom burden and treatment monitoring	Non-invasive, reproducible, quantitative, suitable for repeated use	Symptom-based only; cannot confirm objective gastric emptyng
Upper endoscopy / CT or MRI	Rule out mechanical obstruction and structural/mucosal pathology	Direct visualization of the upper GI tract; biopsy capability	No direct assessment of gastric motility; may miss functional disorders
Gastric emptying scintigraphy (GES)	First-line objective test for confirming delayed gastric emptying and severity stratification	Gold standard; validated retention thresholds; severity classification	Limited availability; radiation exposure; protocol variability; influenced by acute hyperglycemia
^13^C breath test	Alternative or repeat objective assessment, especially when repeated measurements are needed	Radiation-free, repeatable, well tolerated; safe in pregnancy, breastfeeding, children	Indirect measure; influenced by hepatic/pulmonary/small-intestinal factors; need standardization
Second-level diagnostic assessments			
Wireless motility capsule (WMC)	Assessment of multiregional motility (gastric, small-bowel, colonic in selected cases)	Single-test assessment of gastric, small-bowel and colonic transit; radiation-free; well-tolerated in children	May underestimate delayed emptying with severe hypocontractility; different thresholds from GES; cost and limited availability
Gastric ultrasound	Bedside or perioperative evaluation of gastric volume and antral motility	Non‑invasive, radiation‑free, low‑cost, bedside tool	Operator‑dependent; limited standardization; less reliable for solids and half‑emptying time
Antro-duodenal-jejunal manometry	Detailed evaluation of motor patterns in complex or mixed‑type suspected cases	High‑resolution motor characterization; distinguishes neuropathic vs. myopathic patterns	Invasive, technically challenging, limited to specialized centres
Electrogastrography (EGG), including high-resolution mapping	Research and selected phenotyping of gastric dysrhythmias and propagation patterns	Non‑invasive; assessment of slow‑wave frequency, amplitude, and propagation	Largely investigational in routine practice; interpretation requires expertise

Abbreviations: GCSI, Gastroparesis Cardinal Symptom Index; PAGI‑SYM, Patient Assessment of Gastrointestinal Disorders–Symptom Severity Index; GES, gastric emptying scintigraphy; WMC, wireless motility capsule; CT, computed tomography; MRI, magnetic resonance imaging

## Clinical impact and management of insulin therapy

### Clinical impact

The interplay between autonomic neuropathy, impaired enteric neurotransmission, and loss or dysfunction of interstitial cells of Cajal produces a motility disorder that disrupts the temporal coordination between meal ingestion, gastric processing, intestinal absorption, and insulin pharmacodynamics. As a consequence, gastroparesis profoundly affects postprandial glycemic excursions, insulin requirements, and the overall management of diabetes [15, 19, 23, 88, 89]. In diabetic subjects without gastroparesis, prandial insulin is typically administered 10–15 min before a meal to align insulin peak action with carbohydrate absorption into the duodenum and proximal jejunum. allowing efficient postprandial control [90].

In DGP, gastric emptying becomes both delayed and erratic; as a consequence, the timing of nutrient arrival in the small intestine may be hours after food intake and highly variable between meals [91]. Even minimal fluctuations in gastric motility can therefore produce substantial differences in the timing and magnitude of postprandial hyperglycemia [92, 93].

This results in a marked temporal mismatch between insulin action and nutrient absorption [94]. The clinical consequences are well recognized:

1. early postprandial hypoglycemia: Insulin acts before sufficient carbohydrates enter the small intestine; plasma insulin concentrations rise while circulating glucose remains low due to delayed gastric transit [64, 94, 95].
2. late postprandial hyperglycemia: Once nutrients finally reach the absorptive surface of the small intestine, the circulating insulin concentration has declined, leading to a disproportionate and often prolonged glycemic rise [64, 94, 95].

These fluctuations lead to pronounced glycemic variability, and the altered rates of gastric emptying can hinder the ability to appropriately adjust prandial insulin doses to the actual amount of ingested nutrients [96]. Continuous glucose monitoring (CGM) consistently reveals sharp oscillations, delayed peaks, and late postprandial waves of hyperglycaemia alternating with unpredictable hypoglycaemic drops [97].

This glycaemic variability, is associated with both microvascular and macrovascular complications, with reduced quality of life, and increasing in the psychological burden of diabetes management [98–100]. It also complicates both the achievement of recommended glycated hemoglobin (HbA1c) and time in range (TIR) targets [101, 102].

Furthermore, chronic and acute hyperglycemia inhibit gastric motility by reducing vagal cholinergic activity, impairing fundic relaxation, slowing antral peristalsis, and diminishing pyloric compliance, creating a self-perpetuating cycle between worsening metabolic control and gastrointestinal symptoms [6, 23, 92, 103, 104].

Nutritional morbidities are also common: nausea, vomiting, early satiety, and postprandial fullness limit food intake and often lead to involuntary weight loss, micronutrient deficiencies (particularly iron, vitamin B12, vitamin D, and fat-soluble vitamins), reduced protein intake, sarcopenia, increased risk of dehydration and hospitalization [105–108]. Therefore, the clinical impact of gastroparesis extends beyond isolated gastrointestinal dysfunction and must be understood as a multisystem condition globally influencing the course of diabetes and worsening the patient’s overall prognosis.

### Management of insulin therapy

#### Blood glucose control

Optimizing insulin therapy in individuals with DGP requires an individual, adaptive and CGM-guided approach, as delayed and unpredictable gastric emptying prevents a single universal algorithm [3, 14]. The main aim is to minimize early postprandial hypoglycemia and late hyperglycemia while maintaining safe glycemic control.

In patients on multiple daily injections (MDI), administering rapid‑acting insulin after the meal starts, rather than 10–15 min before, can reduce early hypoglycemia but may lead to early postprandial hyperglycemia if gastric emptying is only mildly delayed or if the meal contains rapidly absorbed liquids [109–111]. Splitting the bolus (e.g., part at mealtime and part 30–60 min later) may improve alignment between insulin and nutrient appearance, particularly when supported by CGM trends [92, 112].

In consistently slow gastric emptying, switching from rapid-acting insulin analogues to regular human insulin may be beneficial, as its slower onset (30–60 min) and longer duration (6–8 h) can better match the delayed absorption of carbohydrates [3, 14, 92, 113, 114]. However, this approach increases the risk of hypoglycemia if emptying accelerates unpredictably or hyperglycemia if the delay worsens [113, 114].

#### Dietary and nutritional strategies

Nutritional intervention stabilizes gastric transit and carbohydrate absorption. First‑line strategies include:

- Small, frequent meals (4–6 per day) to reduce gastric distension and symptom burden [105, 115–117];
- Low‑fat and low‑fiber diets, as dietary fat and insoluble fiber delay gastric emptying and exacerbate symptoms [105, 108, 115, 116];
- Liquid or homogenized foods, which are better tolerated than solids [105, 108, 115, 116];
- Small‑particle size diets, which have been shown to improve symptom severity and quality of life compared with a standard soft diet, despite similar effects on gastric emptying [118].

In severe forms, jejunal enteral nutrition may be indicated when oral intake is insufficient or poorly tolerated, bypassing the dysfunctional stomach and improving nutrient delivery and symptom control while parenteral nutrition is reserved for refractory [3, 19, 108].

Nutritional and insulin strategies should be integrated, with CGM‑guided adjustments, to break the vicious cycle between gastric dysmotility and glycemic imbalance.

#### Use of medication in DGP

Concomitant medications must be considered when adjusting insulin therapy. Prokinetics, such as metoclopramide and erythromycin, modifies emptying times and may require earlier or split bolus dosing to avoid early hypoglycemia [2, 3, 14, 119, 120], although their efficacy is often limited and long-term use requires careful monitorg for adverse effects [2, 14, 120].

Metoclopramide, the only drug approved by the FDA for gastroparesis, is limited to < 12 weeks due to extrapyramidal and tardive dyskinesia risk [3, 14, 120, 121], (although an intranasal formulation of metoclopramide was designed to help alleviate symptoms [64, 122]), whereas Domperidone and Erythromycin require careful cardiac monitoring [3, 14, 123].

The selective 5‑HT4 receptor agonist prucalopride may improve nausea, early satiety, bloating, and quality of life in selected patients, though it carries gastrointestinal side effects and requires individualization [49, 124, 125].

Antiemetics such as 5‑HT3 receptor antagonists (e.g., ondansetron) and dopamine‑D2/D3 antagonists (e.g., prochlorperazine) are commonly used to control nausea and vomiting, often in combination with prokinetics [49, 61, 124].

GLP‑1 receptor agonists slow gastric emptying and may worsen gastroparetic symptoms and late hyperglycemia if bolus timing is not adjusted [2, 92–127]; therefore, dose reduction, discontinuation or switching therapies, may be appropriate in symptomatic DGP [14, 126, 127].

### Endoscopic, surgical and device-based interventions

For medically refractory DGP, advanced options include:

- Gastric electrical stimulation (GES), which may reduce vomiting and improve quality of life and glycemic variability in selected patients, although data are largely observational [128];
- G‑POEM (gastric per‑oral endoscopic myotomy), which reduce pyloric resistance improve symptoms in refractory DGP, with success rates around 70% at 1 year in small series [129, 130];
- Laparoscopic pyloromyotomy, reserved for selected patients with focal pyloric dysfunction, although there are limited long-term outcome data [3, 14].

These interventions are reserved for severe, refractory cases and should be performed in centers with motility expertise and multidisciplinary follow‑up.

Table 2 summarizes the main therapeutic strategies emphasizing insulin modulation, nutrition, and the use of prokinetic drugs or advanced technological systems. Table 3 reports key studies on insulin-glucose dynamics in delated and unpredictable carbohydrate absorption.

**Table 2:** Therapeutic strategies in diabetic gastroparesis (DGP)

Category	Strategy	Objective / Mechanism / Clinical Rationale
Prokinetic drugs	**Metoclopramide**	Dopamine D2 receptor antagonist with additional 5-HT4 agonist activity; enhances antral contractions, increases lower esophageal sphincter tone, and accelerates gastric emptying. It reduces nausea and vomiting but is limited by central nervous system adverse effects (extrapyramidal symptoms, tardive dyskinesia), restricting long-term use [120, 121]
	**Domperidone**	Peripheral D2 receptor antagonist that improves gastric motility and reduces symptoms with a lower risk of central adverse effects due to minimal blood–brain barrier penetration. Its use is limited in some countries because of concerns regarding QT prolongation and cardiac arrhythmias [120, 123]
	**Erythromycin**	Gastric emptying, particularly effective in the short term. Tachyphylaxis develops rapidly, limiting long-term efficacy [3, 14]
Nutrition	**Dietary intervention**: small, frequent meals; liquid or semi-solid textures; low-fat and low-fiber content	Reduces gastric distension and symptom burden (nausea, early satiety, bloating). Small-particle and liquid meals empty more rapidly from the stomach, improving symptom control and partially stabilizing postprandial glycemic excursions by reducing the variability of nutrient delivery to the intestine [105, 115–117].
	**Artificial nutrition** post-pyloric enteral feeding (nasojejunal tube or jejunostomy); parenteral nutrition	Ensures adequate caloric and micronutrient intake in patients with severe or refractory gastroparesis and poor oral tolerance. Jejunal feeding bypasses the dysfunctional stomach, improving nutrient absorption and glycemic predictability. Parenteral nutrition is reserved for exceptional cases due to higher risks of infection, thrombosis, and metabolic complications [46, 52, 109].
Insulin therapy and metabolic monitoring	**MDI** delayed bolus, bolus fractionation, use of regular insulin	Attempts to better align insulin action with delayed and unpredictable carbohydrate absorption. Delaying or splitting the bolus reduces early hypoglycemia, while regular insulin, owing to its slower onset and longer duration, may better match late glucose appearance, although variability remains high [3, 14, 92, 113, 114].
	**CGM**	Enables real-time detection of glycemic excursions, particularly postprandial hyperglycemia and delayed hypoglycemia. Provides metrics such as time in range, time in hypoglycemia, and glycemic variability, which are essential for dynamic insulin adjustment in gastroparesis [133, 135–138].
	**CSII**	Allows flexible and programmable insulin delivery tailored to delayed gastric emptying. Extended bolus profiles distribute insulin over several hours, reducing late postprandial hyperglycemia and overall glycemic variability compared with fixed bolus injections [135, 136]
	**AID**	Combine CGM, insulin pumps, and predictive algorithms to automatically adjust basal insulin and correction doses. These systems improve time in range and reduce hypoglycemia in the context of highly variable gastric emptying, although manual meal-related adjustments remain necessary due to delayed carbohydrate absorption and insulin pharmacokinetics [158–161].

Abbreviations: MDI: Multiple Daily Injections; CGM: Continuous Glucose Monitoring; CSII: Continuous Subcutaneous Insulin Infusion; AID: Automated Insulin Delivery

**Table 3:** Key studies relevant to insulin therapy and metabolic mechanisms in diabetic gastroparesis (DPG)

Type of study	Intervention/focus	Clinical relevance for DPG	Year	Ref.
Narrative review	Overview of pathophysiology, diagnosis, treatment	Provides foundational context for understanding mechanisms that underlie insulin–carbohydrate mismatch	2019	[19]
Prospective study	Gastric emptying and meal glucose peak	Demonstrates direct link between emptying time and glucose kinetics, relevant to bolus timing	2018	[91]
Narrative review	Normal and disordered gastric emptying	Integrates pathophysiology and clinical management, supporting adaptive insulin strategies	2022	[92]
Narrative review	Gastric emptying–glycaemia interaction	Provides biological rationale for individualized insulin timing strategies	2015	[93]
Narrative review	PK/PD of rapid-acting insulin analogues	Explains mismatch between insulin action and delayed carbohydrate absorption in DGP	2012	[94]
Experimental physiological study	Gastric emptying and oral glucose tolerance	Foundational evidence for insulin–carbohydrate mismatch	1993	[95]
Observational physiological study	Bidirectional glucose–gastric emptying relationship	Underline vicious cycle between dysmotility and poor glycaemic control	2018	[96]
Post-hoc analysis of RCT	Glycaemic variability and complications	Highlights clinical risk of variability induced by DGP	2020	[98]
Systematic review and meta-analysis	Glycaemic variability and outcomes	Variability associated with adverse outcomes	2022	[99]
CGM-based observational study	High variability alters HbA1c–TIR relationship	Supports CGM-guided insulin adjustments in DGP	2020	[184]
RCT	Pre- vs. postprandial insulin lispro	Supports delayed bolus strategies in conditions of delayed gastric emptying	2004	[109]
Open-label prospective pilot study	Diabetic patients with gastroparesis and Sensor-augmented CSII	Significant reduction in hypoglycaemia, improved HbA1c and time-in-range; safer insulin titration despite unpredictable gastric emptying	2018	[135]
Observational study	CSII vs. previous MDI	Improved glycaemic control and reduction in severe hypoglycaemia; better matching of insulin delivery with delayed gastric emptying	2011	[136]
Proof-of-concept observational study	CGM-derived glycaemic variability metrics	Glycaemic variability patterns could discriminate gastroparesis, supporting CGM use for detection and monitoring	2021	[145]
Observational study	Diabetic patients with gastroparesis and CGM	CGM revealed marked postprandial hyperglycaemia and delayed glucose peaks not detected by SMBG, highlighting insulin–nutrient mismatch	2011	[146]
Case series	Adults with T1D, gastroparesis and Hybrid closed-loop system	Feasibility and safety of automated insulin delivery in gastroparesis, with improved glucose stability	2021	[153]
Observational study	Adults with T1D, gastroparesis and Hybrid closed-loop system	Improved time-in-range and reduced glycaemic excursions without increased hypoglycaemia	2019	[154]

Abbreviations: DPG: Diabetic GastroParesis; RCT: Randomized Controlled Trials; PK: Pharmacokinetcs; PD: Pharmacodybamics; T2D: Type 2 Diabetes; T1D: Type 1 Diabetes; CGM: Continuous Glucose Monitoring; CSII: Continuous Subcutaneous Insulin Infusion; MDI: Multiple Daily Injections; SMBG: Self-Monitoring Blood Glucose

## The role of technology in clinical management

Digital innovations in glucose sensing and insulin delivery have improved glycemic management in diabetes and offer particular advantages in patients with DGP, where delayed and variable carbohydrate absorption leads to erratic glycemic excursions [131, 132].

In this context, CGM plays a central role by enabling visualization of glycemic trends and revealing patterns that are missed with capillary glucose testing [132, 133]. CGM helps characterize the timing, amplitude, and frequency of excursions [133–139], and provides quantitative metrics such as TIR, time in hypoglycemia (TBR) and hyperglycemia (TAR), and indices of glycemic variability, which are increasingly used as clinically meaningful targets in diabetes management [140–144]. These data facilitate a dynamic, iterative optimization of insulin dosing, bolus timing, and fractionation, moving away from purely empirical adjustments [132, 143, 145, 146].

The integration of CGM with continuous subcutaneous insulin infusion (CSII) expands therapeutic possibilities, allowing for more precise and safer glycemic control, with possible improvements in HbA1c, gastrointestinal symptoms, tolerance to liquid meals and quality of life [135, 136, 147]. Sensor‑augmented pump systems (SAP) enable adaptive insulin delivery, including dual‑wave or square‑wave boluses, which distribute insulin over several hours and better match the slow and prolonged glucose absorption typical of DGP [54, 112, 132, 148, 149], particularly in high‑fat or high‑protein meals [150–152].

Furthermore, hybrid closed‑loop systems (Automated Insulin Delivery, AID) have been increasingly explored in DGP [3, 14, 131, 132]. These devices combine an insulin pump and CGM with predictive algorithms that adjust insulin delivery in real time. Avaiable data suggest that such systems can increase TIR and reduce hypoglycemia in patients with DGP, with improvements comparable to those observed in individuals without gastrointestinal complications, although data remain limited and largely observational [153–157].

However, current algorithms are primarily responsive to immediate glycemic changes and may not fully compensate for delayed carbohydrate absorption, especially from meals rich in fat and protein that can induce delayed hyperglycemia [131, 132, 158]. For these reasons, active patient engagement and clinician oversight remain essential, and manual interventions such as meal announcement, extended bolus programming, and corrective dosing continue to play a key role in optimal management [158–161].

## Future prospects

The management of DGP is moving toward an integrated, mechanism-driven approach that combines pharmacological innovation, advanced digital technologies, and regenerative medicine, with the goal of mitigating the fundamental temporal mismatch between insulin action and nutrient absorption [14, 15, 25, 119].

In this context, glucose-responsive insulins (GRIs) and controlled-release insulin analogues aim to decouple metabolic efficacy from rigid injection timing, modulating insulin activity in response to ambient glucose levels: GRIs are designed to increase their activity or release rate during hyperglycemia and to reduce activity during normoglycemia or impending hypoglycemia [162–164]. However, these agents remain in early stages of development, being used only in preclinical models [165, 166].

In parallel, the evolution of insulin delivery systems is expected to further improve glycemic control [163, 167]. The incorporation of artificial intelligence (AI) and machine-learning approaches into automated insulin delivery systems is currently being developed and may integrate multimodal data streams, including CGM trends, historical meal responses, dietary composition, insulin-on-board, and contextual clinical variables, to dynamically infer patterns of delayed or prolonged glucose appearance and adjust insulin delivery accordingly. In patients with DGP, algorithms could “learn” the individual’s gastric emptying behavior over time and reducing both hypoglycemia and late postprandial hyperglycemia [159, 167–170]. Although these systems are under active investigation, prospective validation specifically in patients with DGP remains limited, and their effectiveness in this setting requires further study [171].

At the same time, regenerative strategies targeting ICCs and enteric neuronal networks seek to correct the cause of delated gastric emptying [56, 172, 173]. ICC survival and network integrity depend on tightly regulated signaling pathways, particularly the SCF/c-KIT signal, which regulates ICC proliferation and function [56, 174, 175]. In pre-clinic studies, modulation of the SCF/c-KIT pathway, has been shown to promote ICC survival, reduce apoptosis, and improve gastric motor function [174, 175].

Regenerative approaches are therefore being explored along multiple, complementary lines, including the differentiation of stem or progenitor cells into ICC-like phenotypes [176, 177], pharmacological stimulation of endogenous ICC proliferation or survival through growth factor modulation [178–180], and the engineering of biointegrated tissue constructs that combine ICCs with enteric neurons to restore coordinated pacemaker activity and neuromuscular transmission [179, 181, 182]. These strategies represent a conceptual shift toward disease-modifying interventions althought their translation into safe and effective clinical therapies will require substantial further research [183] .

The three approaches described are not alternatives but complementary. Whitin a future precision-medicine framework, a patient with gastroparesis could be accurately characterized, receive “smart” insulin therapy (GRI or adaptive AID) to stabilize blood glucose, and, if refractory, potentially be a candidate for targeted regenerative strategies that restore gastric pacemaker function.

## Conclusions

DGP significantly complicates insulin management, requiring an individualized approach that considers the variability of gastric emptying and its impact on glycemic patterns. The choice and timing of insulin, combined with nutritional and pharmacological intervention, and with advanced technologies, should be adapted case by case based on symptom patterns, and meal characteristics, and CGM-derived metrics.

The technologies available are reshaping the therapeutic landscape and offer the potential to reduce glycemic variability and treatment burden. However, robust clinical evidence specifically addressing patients with DGP remains limited, highlighting the need for dedicated prospective studies and multidisciplinary management models. The ultimate goal is to improve metabolic control and, above all, to improve patients’ quality of life by reducing the clinical and psychological burden of a complication that profoundly affects the daily life and prognosis of people with diabetes.

## Supplementary Information

Below is the link to the electronic supplementary material.

Supplementary Material 1

## Data Availability

No datasets were generated or analysed during the current study.
