# Supplementary material for: Diabetic gastroparesis: pathophysiology and impact on insulin timing choices
Source: Endocrine. 2026 Jun 23;91(1):211. doi: 10.1007/s12020-026-04693-6 (PMC13290835; doi:10.1007/s12020-026-04693-6)
Supplement: Supplementary file 1 — Supplementary Material 1 [file 12020_2026_4693_MOESM1_ESM.docx]

***Appendix S1.*** *PubMed search strategies*

Diabetic autonomic neuropathy

**("Gastroparesis"[Mesh] OR gastroparesis OR "Gastric Stasis") AND ("Diabetes Mellitus"[Mesh] OR diabetes OR diabetic) AND ("Diabetic Autonomic Neuropathy" OR "Diabetic Neuropathies"[Mesh] OR "Autonomic Neuropathy" OR "Enteric Neuropathy" OR "Gastric Motility").**

Diagnosis

**("Gastroparesis"[Mesh] OR gastroparesis OR "Gastric Stasis") AND ("Insulin"[Mesh] OR insulin timing OR "postprandial glucose" OR hypoglycemia).**

Therapy

**(“Gastroparesis” [Mesh] OR gastroparesis OR “Gastric Stasis”) AND (“Continuous Glucose Monitoring” OR CGM OR “Insulin Pump” OR CSII OR “Closed-Loop Systems” OR “Artificial Pancreas”).**

**("Gastroparesis"[Mesh] OR gastroparesis OR "Gastric Stasis") AND ("Diabetes Mellitus"[Mesh] OR diabetes OR diabetic) AND ("Diabetic Autonomic Neuropathy" OR "Diabetic Neuropathies"[Mesh] OR "Autonomic Neuropathy" OR "Enteric Neuropathy" OR "Gastric Motility") diet OR nutrition OR feeding).**
